# Weight-related bullying in schools: a review of school anti-bullying policies

**DOI:** 10.1186/s12889-025-23170-9

**Published:** 2025-05-30

**Authors:** Amanda Hughes, Elisabeth Grey, Alice Haigherty, Fayth Shepherd, Fiona Gillison, Georgie MacArthur, Caoimhe Gowran, Rebecca Langford

**Affiliations:** 1 Medical Research Council Integrative Epidemiology Unit, Bristol Medical School, University of Bristol, Oakfield House, Oakfield Grove, Bristol, BS8 2BN UK; 2 The National Institute for Health and Care Research Applied Research Collaboration West (NIHR ARC West), University Hospitals Bristol and Weston NHS Foundation Trust, 9th Floor, Whitefriars, Lewins Mead, Bristol, BS1 2NT UK; 3 Department of Psychology, University of Bath, Claverton Down, Bath, BA2 7AY UK; 4 Centre for Motivation and Behaviour Change, Department for Health, University of Bath, Bath, BA2 7AY UK; 5 North Somerset Council, Town Hall, Walliscote Grove Road, Weston-super-Mare, BS23 1UJ UK; 6 Bristol City Council, City Hall, PO Box 3399, Bristol, BS1 9NE UK; 7 Centre for Public Health, Bristol Medical School, University of Bristol, Canynge Hall, 39 Whatley Rd, Bristol, BS8 2PS UK

**Keywords:** Bullying, School, Weight, Obesity, Stigma, Bias, Victimisation, Policy, UK

## Abstract

**Introduction:**

Weight is reported to be the most common target for bullying at school - far more common than other targets such as ethnicity, religion or sexual orientation. Research suggests weight bias and stigma - including negative beliefs, attitudes and discriminatory behaviours related to a person’s weight - is prevalent in educational settings among both pupils and staff. Most schools have anti-bullying policies. Best practice recommendations advise policies should explicitly identify forms of unacceptable behaviour, such as racism or homophobia. We conducted an audit of secondary schools in southwest England to determine if/how they mention weight-related bullying in their policies, and whether this differs by school-level factors.

**Methods:**

We obtained lists of all secondary mainstream state, private, and special schools in seven local authorities and downloaded anti-bullying policies from their websites. Policies were searched for key words related to weight and size. We also recorded whether policies mentioned appearance or other key targets for bullying, such as race, religion, sexuality etc. We obtained school level data including size, gender mix, academic performance and quality ratings.

**Results:**

From 255 schools with an available bullying or behaviour policy, only 6.7% specifically mentioned weight-related bullying. Just under half (48.6%) mentioned bullying in relation to appearance. Bullying was most often mentioned in relation to race/ethnicity (94.5%), sexual orientation (93.3%), gender (85.9%), religion (84.9%) or gender identity (67.5%). Private schools (*N* = 40) were more likely to mention weight-related bullying (17.5%) than mainstream state schools (*N* = 148, 6.1%). No special schools, whether state (*N* = 41) or private (*N* = 26), mentioned weight-related bullying in their policies. There was no strong evidence that other school characteristics made a difference, but small numbers limited statistical power of these comparisons.

**Conclusion:**

There is a significant mismatch between the prevalence of weight-related bullying in schools and its representation within school anti-bullying policies. Some types of school are more likely than others to mention weight-related bullying in their policies. We recommend that schools explicitly recognise weight-related bullying in their anti-bullying policies and explore how to support staff and pupils to take action.

**Supplementary Information:**

The online version contains supplementary material available at 10.1186/s12889-025-23170-9.

## Introduction

Bullying is a significant public health issue [1], defined as involving intentional harm, repeated over time, and usually accompanied by an imbalance of power [2, 3]. In England and Wales, over a third (35%) of 10–15 year-olds have experienced a face-to-face bullying event, while a fifth (19%) have reported experiencing cyber bullying (the latter being more common in girls, 23% vs. 16%) [4]. Both in-person (68%) and cyber bullying (65%) were most commonly perpetrated by someone at the victim’s school [4]. Bullying is most prevalent in early adolescence between ages 11–13 [4, 5], coinciding with the start of secondary school in the UK.

Children with overweight or obesity are more likely to be bullied than their peers with a healthy weight [6]. More than half of young people (55%) have been bullied because of their appearance, most commonly because of their weight [7]. A cross-national survey identified bullying for ‘being fat’ as the most common reason for youth bullying, far ahead of other reasons such as ethnicity, religion or sexual orientation [8]. In a recent UK survey of adolescents, ‘attitudes towards my appearance’ (including weight) was similarly reported as the number one reason for being bullied [9]. Nearly half (47%) of participants who had been bullied identified this as the reason, in comparison to 11% for sexual orientation, 6% for race, 5% for religion and 4% for gender identity. The prevalence of underweight children in the UK is low (2% underweight vs. 36% with overweight or obesity) [10]. However, while much less research has looked at the association between underweight and bullying [6], some evidence suggests that underweight status puts children, particularly boys, at greater risk of bullying than healthy weight peers [11].

Bullying can have lasting effects on an individual’s mental and physical health. Children and young people with obesity who experience weight-related bulling are 2–3 times more likely to report suicidal intent or engage in self-harming behaviour [12]. Research suggests experiencing weight-related bullying can lead to additional weight gain over time [13, 14]. In addition, research suggests weight stigma can negatively impact educational outcomes [15–17].

In the UK, state-funded schools are required by law to have a policy in place that includes measures to prevent bullying behaviour [3, 18]. The same does not legally apply to private schools, though many will have an anti-bullying or behaviour management policy in place. A meta-analysis by Lee and colleagues [19] found that interventions which included establishing a school policy on bullying were associated with greater reductions in victimisation than those that did not. Evidence from the US suggests school district mandates and laws around bullying can decrease the probability of bullying in schools [20, 21].

The Anti-Bullying Alliance is a national charity made up of individuals and organisations working to stop bullying and influence policy in England, Wales and Northern Ireland [22]. The Anti-Bullying Alliance states ‘*a good [bullying] policy ensures pupils and staff are clear on expectations and on how you tackle bullying as a community.’* [18] This charity provides support to schools on writing school bullying policies based on a series of guiding principles. These include *‘agree as a school community what you mean by bullying’* and *‘list the different behaviours that bullying might include’*. It also suggests schools should specifically reference *‘behaviour that is homophobic*,* transphobic*,* racist*,* targeted at faith*,* sexist and disablist.’* It is notable that this document includes no reference to weight, size or appearance.

Weight-related bias and discrimination has been described as a socially acceptable form of stigma [23, 24]. Negative views of people with obesity are commonplace in the UK. Research has found ‘unequivocal evidence’ of weight stigma within UK national newspapers [25]. National surveys have further found evidence of both implicit and explicit anti-fat attitudes in UK adults across demographic groups [26, 27]. For example, in a nationally representative 2015 survey [28], around 28% of British adults agreed that ‘most very overweight people are lazy’. Negative views of people with underweight status are also prevalent [29], particularly of individuals perceived to have an eating disorder such as anorexia nervosa [30, 31].

Negative attitudes about people with obesity have also been found to be prevalent within educational settings, with discriminatory views found among pupils, as well as pre- and in-service teachers [32]. Despite the known harms of bullying and the apparent ubiquity of weight-related bullying, efforts to tackle weight-related bullying and stigma in schools remain under-developed. A recent review of reviews of school-based bullying interventions identified 275 studies, but none of these explicitly addressed weight-related bullying [33]. Research from both the UK and US suggest weight-related bullying may be under-represented in school anti-bullying policies [34, 35].

In this study we explored whether and to what extent weight-related bullying is explicitly acknowledged within school anti-bullying policies, and whether this varies by school-level factors, focusing on secondary schools within a sample of seven south-west local authorities in the UK.

## Method

### Study design

Policy content review of school anti-bullying policies, publicly available on school websites.

### Public involvement

We discussed this project with members of Bristol’s Young People’s Advisory Group (YPAG) when planning the study (*N* = 5, 3f) and during interpretation of the results (*N* = 6, 4f; some of whom had been at the first meeting). YPAG members were aged between 10 and 16 years. They advised us that weight-related bulling was common in schools (particularly secondary schools) and generally ‘tolerated’ by school staff and dismissed as ‘banter’. Making derogatory comments about someone’s weight would likely result in no action; they contrasted this to racist comments which would likely be met with immediate suspension. These discussions informed (a) our focus on secondary schools and (b) the types of bullying we included in our data extraction (e.g. racism). We refer to their comments on the findings in the discussion.

### Study area

We focused our policy review on the seven local authorities within the NIHR Applied Research Collaboration West (ARC West) region: Gloucestershire, South Gloucestershire, Bristol, North Somerset, Bath and North East Somerset, Wiltshire and Swindon. Though all in one region, this area includes city, town and rural areas and variation in deprivation levels (Table 1).

**Table 1:** Characteristics of local authorities in the study

	Rural-urban classification	Income deprivation rate quintile (1 = most deprived, 5 = least deprived)	Percentage of pupils achieving grades 4 (pass) or above in English and Mathematics GCSEs (2022/23)	Percentage of Year 6 (10–11 yrs) with overweight or obesity
England	n/a	n/a	65.40%	36.6%
Southwest	n/a	n/a	65.60%	32.9%
Bath and North East Somerset	Urban with Significant Rural	4	72.10%	29.6%
Bristol, City of	Urban with City and Town	2	63.40%	34.9%
Gloucestershire	Urban with City and Town	2	70.80%	34.1%
North Somerset	Urban with Significant Rural	3	66.90%	31.6%
South Gloucestershire	Urban with City and Town	5	64.30%	32.1%
Swindon	Urban with City and Town	3	63.50%	36.5%
Wiltshire	Largely Rural	4	66.90%	32.7%

**Rural-urban classification data from**: https://www.gov.uk/government/statistics/2011-rural-urban-classification-of-local-authority-and-other-higher-level-geographies-for-statistical-purposes

**Income data from**: https://cy.ons.gov.uk/peoplepopulationandcommunity/personalandhouseholdfinances/incomeandwealth/datasets/mappingincomedeprivationatalocalauthoritylevel

**Education data from**: https://explore-education-statistics.service.gov.uk/data-tables

**Obesity data from**: https://digital.nhs.uk/data-and-information/publications/statistical/national-child-measurement-programme/2022-23-school-year

Educational performance varies within these local authorities, including three with below average performance (Bristol, South Gloucestershire and Swindon) and four with above average performance (Bath and North East Somerset, Wiltshire, Gloucestershire, and North Somerset), as measured by the percentage of pupils achieving grade 4 (pass) or above in English and Maths GCSEs (exams taken at the end of secondary school) (Table 1).

Child obesity levels (measured in the final year of primary school, aged 10–11 years) are lower in all seven local authorities than the national average for England, though three local authorities (LAs) have higher rates than the regional southwest average (Bristol, Gloucestershire and Swindon) (Table 1).

### School selection

Within the UK, most local authorities operate a two-tier school system of primary schools (age 4–11) and secondary schools (11–16+). We focussed our analysis on secondary schools following input from our YPAG. Box 1 explains the three broad categories of schools in the UK.

**Box 1:** Types of schools in the UK

State mainstream schools	Receive funding through their local authority or directly from the government. Types of state mainstream school include: - Local Authority (LA) maintained– these are funded and overseen by the local authority, follow the national curriculum, and are not affiliated with religious groups. - Academies– these are converted LA maintained schools that are now run by academy trusts. They do not have to follow the national curriculum. - Free schools– these are new schools (not converted LA maintained schools) and run on a not-for-profit basis and do not use academic selection processes and do not have to follow the national curriculum.
Private schools	Also known as ‘independent schools’. They are not funded by the government and instead charge fees. They do not have to follow the national curriculum.
Special schools	These schools are for pupils with special educational needs. They can be state or privately funded.
Information from: https://www.gov.uk/types-of-school/academies and https://commonslibrary.parliament.uk/research-briefings/sn07059/	

We aimed to include all state mainstream, private, and special secondary schools (which can be state or privately funded), obtaining a list of all eligible schools from government and local authority websites and the Independent Schools Council website. If a school included both primary and secondary age children (more common in private and specialist settings), they were included in our analysis.

### Data extraction

School background data for state-funded schools (mainstream and special) were downloaded from a UK government education website [36]. Data for private (non-special) schools were obtained from the Independent Schools Council [37].

We downloaded anti-bullying policies directly from school websites. If a school did not have a specific anti-bullying policy but discussed bullying within their ‘behaviour’ policy, we extracted data from this. Where schools did not have their own anti-bullying policy, but referred to a trust-level policy, we extracted data from the trust policy. If we could not locate a relevant policy on the school’s website, we emailed the school to request a copy. If we received no response, we recorded this as missing data.

For each school, we recorded whether policies mentioned weight as a possible target for bullying. In some cases, policies used terms such as size or shape, rather than weight specifically; while this could refer to any size or shape body, we felt this was likely to include people with overweight or obesity and recorded this as a positive response. We also separately recorded if policies mentioned appearance more generally in their policy. Where policies referred to weight (or size/shape) or appearance, we extracted the page number and a direct quote from the policy. We also recorded whether policies mentioned other common targets for bullying, selecting five such categories based on our public involvement conversations with young people: these were race/ethnicity, religion, gender, sexual orientation, and gender identity. We recorded any other named targets of bullying and identified which were mentioned by at least 5% of schools (Supplementary materials).

Policies were searched using the search function (Ctrl + F) using a list of key words developed through discussion among co-authors (Table 3). Care was taken when identifying key words in the documents to ensure they were referring to bullying. (For example, *‘breaches of academy rules on uniform or****appearance’*** is clearly unrelated to bullying.)

For all schools, we considered the following predictors of whether a school mentioned weight-related bullying in their policy: the type of school (state mainstream, private or special), whether it was mixed or single sex, the age range of pupils, and whether the school had a specific bullying policy or a more general behaviour policy. Number of pupils was dichotomized for analysis as below 500 (38.4%) or 500 or more (59.2%), following visual inspection of the distribution. Within mainstream state schools, we also considered whether the school was a Local Authority maintained school or an academy or free school (which have a greater degree of independence than Local Authority schools), and whether the school had a selective admissions policy. Two measures of mainstream school quality were included: (1) OFSTED rating– statutory inspection framework with ratings from 1 (outstanding) to 4 (inadequate) [38] and (2) Progress8 rating– a ‘value-added’ measure indicating how much a school has helped pupils progress, compared to expected levels of improvement [39]. To capture socioeconomic composition of pupils, we considered Index of Multiple Deprivation (IMD) decile based on school postcode, and the percentage of pupils eligible for free school meals; these were categorized as above or below the median for analysis. Within private schools, we captured school quality using the Independent Schools Inspectorate (ISI) ratings for academic attainment and personal development.

A data extraction form was developed by RL and EG and piloted on 20 schools by AHa and FS. These data were independently checked by RL for accuracy. Minor revisions and clarifications were made to the extraction process as a result. AHa and FS extracted all subsequent data, with a further 10% randomly checked by EG/RL.

### Data analysis

The characteristics listed as targets of bullying (including weight) were ranked by how frequently they were mentioned across all schools. Differences between each school-level characteristic and reference to weight-related bullying (yes/no) in a school’s policy were calculated using chi-squared tests, which are useful for exploring differences between groups in datasets with relatively small numbers of observations (*n* = 255). For non-ordered categorical variables, we ran pairwise tests using the largest group as a reference category, and for ordered categorical variables a chi-squared test-for-trend. Because some mainstream state schools used an anti-bullying or behaviour policy set at the trust level, we reran comparisons for school type considering only one school from each of these trusts. All analyses were conducted by AHu.

## Results

Within the seven ARC West local authorities, there were 263 secondary schools, including 148 state mainstream schools, 43 private schools and 72 special schools (45 state funded, 27 privately funded). We were able to obtain bullying or behaviour policies for data extraction for 97% of schools. Eight schools (four state special, three private, and one private special), did not respond to our requests for a copy of their policy, and could not be included. The analytic sample therefore comprised 255 schools, ranging from 9 to 2157 pupils (Table 2).

**Table 2:** Characteristics^a^ of all 255 schools in study sample

	Category	*N*	%
Type	State, mainstream	148	58.0
	State, special	41	16.1
	Private	40	15.7
	Private, special	26	10.2
Mixed or single sex	Mixed	237	92.9
	Boys only	5	2.0
	Girls only	13	5.1
Phase of education	Secondary (11+)	165	64.7
	Begins earlier	90	35.3
Type of policy	Bullying	222	87.1
	Behaviour	32	12.6
	*Missing*	1	0.4
	**Median (IQR)**	**Range**	*N missing*
Number of pupils	765 (164–1178)	9 to 2157	6

^a^ School background data obtained from https://www.compare-school-performance.service.gov.uk/ and https://www.isc.co.uk/. Policy data were obtained from schools’ individual websites

Table 2 shows key characteristics of all schools in our sample. More detailed characteristics relating to mainstream state schools (*N* = 148), special state schools (*N* = 41), private schools (*N* = 40), and private special schools (*N* = 26) are presented in Supplementary Tables 1–4.

## Frequency with which bullying targets were mentioned

Most schools (87%) had a dedicated anti-bullying policy, the remaining 13% referring to bullying within their behaviour policy (Table 2). Most schools had their own school-specific policy, but some (*N* = 29) state schools used a Trust-level policy (Table S1).

Almost all schools mentioned specific targets of bullying (Table 3). The most frequently mentioned were race/ethnicity (94.5%), sexual orientation (93.3%), gender (85.9%), religion (84.7%) and gender identity (67.5%).

**Table 3:** Targets of bullying mentioned in bullying or behaviour policies

Rank	Bullying target	Key search words	*N*	%
1	Race/ethnicity ^a^	Race, racial, racist, ethnicity, ethnic, culture, Black, Asian, BME ^b^ (Black and Minority Ethic), BAME ^b^ (Black, Asian and Minority Ethics), antisemitism/ic, nationality	241	94.5
2	Sexual orientation ^a^	Sex, sexuality, lesbian, gay, bi, orientation, homophobia/ic, LGBT* (lesbian, gay, bisexual and transgender)	238	93.3
3	Gender ^a^	Gender, sex, male, female, sexist, misogyny/ist	219	85.9
4	Religion ^a^	Religion, religious, faith, belief, antisemitism/ic, Islamophobia/c	216	84.7
5	Gender identity ^a^	Gender, sex, non-binary, transphobia, gender reassignment, LGBT, queer, nonbinary	172	67.5
6	Disability	Disability, disabilities, disabled, ableist	137	53.7
7	Appearance	Appearance, look(s)	124	48.6
7	Special educational needs	Special needs, special educational needs, SEN(D) (Special Educational Needs and Disabilities), learning difficulties	124	48.6
8	Carer	Caring, carer	78	30.6
9	Family or home circumstances	Family, home	69	27.1
10	Health	Health, medical	60	23.5
11	In care	In care, cared, looked after	53	20.8
12	Adopted	Adoption, adopted	39	15.3
13	Pregnancy or maternity	Pregnancy, pregnant, maternity, maternal	35	13.7
14	Age	Age, ageism, ageist	35	13.7
15	Socioeconomic	Socioeconomic, socio-economic, economic, class, poverty, income	28	11
16	Weight^†^	Weight, size, fat, body size, body shape	17	6.7
17	Marital status (not in relation to parents)	Marital, marriage, married	16	6.3

^a^ In initial list. Other categories emerged from the data

^b^ Despite recent criticism of these terms [40], we included these acronyms among our search terms since it was plausible that it would be mentioned in school policies (BAME was mentioned)

Only 17 schools (6.7%) mentioned weight, size or shape in their policies in relation to bullying, making weight the 16th most mentioned characteristic in relation to bullying (Table 3). However, appearance-related bullying was mentioned in 48.6% of school policies (Table 2.) Notably, characteristics mentioned much more frequently than weight included being a carer (30.6%), pregnancy or maternity (13.7%) and age (13.7%). Being a carer and pregnancy occur relatively infrequently among children, as compared to the high population prevalence of children with overweight or obesity, and ageism is unlikely to be an issue among young persons.

Of the 17 schools referring to weight-related bullying, only four mentioned weight specifically (alongside size in all four cases), one mentioned size only, and another mentioned shape only (examples provided in Table 4). A further five schools only referenced weight obliquely, giving it as an example of why a pupil might be reluctant to report bullying. (e.g. acknowledging that a child may think ‘It’s my fault for being overweight anyway’).

**Table 4:** Examples of how weight-related and appearance-related bullying were discussed in school policies

**Examples of how weight-related bullying was discussed in school policies**
*“Perceived physical limitations*,* such as size and weight*,* and other body image issues*,* can result in bullying"*
*“The following themes are often found to be used as a basis for the bullying or aggression:… Appearance– size*,* height*,* weight*,* dress*,* personal features”*
*“Bullying may involve actions or comments… which focus on… other physical attributes (such as hair colour or body shape)”*
*“There are many reasons why a student who has suffered bullying may be reluctant to report it. He / She may become demoralised and may think*,* for example… it is all my fault anyway for being overweight / too studious etc”*
**Examples of how appearance-related bullying was discussed in school policies**
*“Bullying can be driven by prejudice or fear of difference. It can be linked to • Race*,* religion or culture • Gender • Sexual orientation • Disability or special need • Long term illness • Appearance • Family arrangements • Any protected characteristic within the Equality Act 2010”*
*“Bullying can take many different forms…. Teasing/ridicule - people laughing at your hair*,* clothes*,* or the way you look”*
*“This policy covers all types of bullying including:… Bullying related to physical appearance”*
*“Forms of bullying […] Bullying related to appearance or physical/mental health conditions”*
*“Discrimination-based bullying: bullying may also be: […] related to a person’s disability*,* special educational needs*,* learning difficulty*,* health or appearance”*
*“Bullying that seeks to hurt people by drawing attention to their body shape*,* hair colour*,* manner of dress*,* alleged sexual attractiveness (or lack of it)*,* close friendships (or the absence of intimate relationships in an individual’s life)”*

In the 124 policies referring to appearance-related bulling, 47 included the term appearance as one in a list of possible targets for bullying (examples provided in Table 4). A further 32 policies included similar descriptions including physical appearance (*N* = 12), personal appearance (*N* = 10), appearance-related differences (*N* = 6) and the way you look (*N* = 4). A further eight policies referred to appearance in relation to other physical characteristics such as clothing, hair (colour), attractiveness or belongings. In 37 policies, appearance was specifically linked to health. Nine policies that mentioned appearance also included reference to weight-related key words (weight, size, body image, body shape); only these policies were scored positively for mentioning weight and were included in the 17 policies referred to above. (NB Numbers total more than 124 as some policies referred to appearance in more than one way.)

### Analysis against school characteristics

Table 5 shows how the likelihood of a school mentioning weight-related bullying in their policies differs by school characteristics. Private (non-special) schools were significantly more likely to mention weight-related bullying in their policies than mainstream state schools (17.5% compared to 6.8%, *p* = 0.04). Restricting the analysis to one school per trust where policy was determined at trust level did not alter conclusions (17.5% vs. 6.6%, *p* = 0.04). No special schools, whether state or private, mentioned weight-related bullying in their policies, and no schools with only a general behavioural policy mentioned weight-related bullying. However, small cell sizes meant these differences could not be distinguished from the null. There were no clear differences between mixed and single-sex schools, schools whose intake begins at secondary or earlier, or size of school (all *p* > 0.05, but with small cell sizes for some groups).

**Table 5:** Predictors of including weight/shape/size in school anti-bullying or behaviour policies

**Across all schools**			
	**Category (*N***)	**% mentions weight**	**chi-squared** ***p*** ^**a**^
Type	State, mainstream (148)	6.8	*Ref*
	State, special (41)	0	0.09
	Private (40)	17.5	0.04
	Private, special (26)	0	0.17
Mixed or single sex	Mixed (237)	6.75	*Ref*
	Boys only (5)	0	0.55
	Girls only (13)	7.7	0.90
Phase of education	Secondary (165)	7.9	0.29
	Begins earlier (90)	4.4	
Type of policy	Bullying (222)	7.7	0.11
	Behaviour (32)	0	
Number of pupils	Less than 500 (98)	6.1	0.72
	500 or more (151)	7.3	
**Mainstream state schools only**			
	**Category**	**% mentions weight**	**chi-squared p** ^**a**^
Type	Academies (122)	5.7	*Ref*
	Free schools (13)	23.1	0.02
	LA maintained (13)	0	0.38
Selective	No (124)	6.45	0.43
	Yes (9)	0	
Ofsted rating	Outstanding (17)	5.9	0.71
	Good (111)	8.1	
	Requires improvement (12)	0	
	Inadequate (3)	0	
Progress8	Well above average (20)	0	0.61
	Above average (29)	10.3	
	Average (64)	4.7	
	Below average (12)	8.3	
	Well below average (13)	7.7	
Level policy set at	School (119)	5.9	0.39
	Trust (29)	10.3	
IMD decile ^b^	Below median (66)	6.1	0.76
	At or above median (82)	7.3	
% of pupils eligible for free school meals ^c^	Below median (71)	2.8	0.06
	At or above median (74)	10.8	
**Private (non-special) schools only**			
	**Category**	**% mentions weight**	chi-squared p*
ISI ^d^ rating– academic attainment	Excellent (26)	19.2	0.21
	Good (7)	0	
ISI^d^ rating– personal development	Excellent (30)	16.7	0.44
	Good (3)	0	

^a^Non-ordered categorical variables: pairwise comparisons with largest group as reference. Ordered categorical variables: chi-squared test-for-trend. ^b^ IMD = index of multiple deprivation (a higher value indicates a greater level of deprivation). Median IMD decile among state mainstream schools = 7. ^c^ median % eligible for free school meals among state mainstream schools = 17.7. ^d^ Independent Schools Inspectorate

Within mainstream state schools, free schools were considerably more likely to mention weight-related bullying than academies (23.1% vs. 5.7%, *p* = 0.02). Restricting analysis to one school per trust where policy was determined at the trust level did not alter conclusions (28.6% vs. 5.9%, *p* = 0.03). No Local Authority maintained schools mentioned weight-related bullying in their policies.

All private (non-special) schools which mentioned weight-related bullying in their policies (*N* = 7) had ‘excellent’ ISI ratings for both academic attainment and personal development. However, small cell sizes meant that differences by ISI ratings were not distinguishable from the null.

No private or state-funded special schools mentioned weight-related bullying in their policies, so no stratified analyses could be run for these school types.

## Discussion

Weight-related bullying is reportedly the most common form of bullying experienced by young people [8]. Across our study area 30–37% of children starting secondary school are living with overweight and obesity, yet our survey of 255 secondary schools in southwest England revealed that only 6.7% of anti-bullying policies specifically mention weight. This is in stark contrast to the frequent specification of other forms of bullying. Compared with weight, school anti-bullying policies were 14 time more likely to mention race/ethnicity or sexual orientation, 13 times more likely to mention gender or religion, and 10 times more likely to mention gender identity.

A similar pattern was found by de Wit’s [34] analysis of 26 London-based schools (13 state and 13 private international schools). Across these 26 policies, bullying in relation to sexuality or gender identity was most frequently mentioned (315 times), followed by references to Special Educational Needs and Disabilities (169 mentions), race (156 mentions) and religion (75 mentions). Bullying in relation to weight or appearance was mentioned just 24 times in total anywhere within the 26 school policies. (de Wit did not provide data on how many of the 26 policies mentioned weight/appearance at least once). These two studies suggest there is a significant mismatch between the lived experience of weight-related bullying in schools and the bullying behaviours specifically prohibited within school anti-bullying policies.

In developing anti-bullying policies, schools appear to have drawn heavily on the protected characteristics outlined in the UK’s 2010 Equality Act which includes, for example, race, sexual orientation, gender, religion, gender identity and disability; 5 schools explicitly mentioned bullying related to protected characteristics. Moreover, in many cases, school policies specified protected characteristics which were surprising in being of less relevant to adolescents and children than they are for adults. For example, twice as many schools mentioned pregnancy as mentioned weight in their anti-bullying policies. The UK conception rate for under 16s is 0.02% [41]; by contrast, over 1 in 3 children entering secondary school in England is living with overweight or obesity [10]). Similarly, roughly the same number of policies specified bullying in relation to marital status (6.3%) as weight (6.7%), despite marriage being illegal for those under 18 years of age in the UK [42].

While specification of weight-related bullying was low, nearly half (48.6%) of policies did mention bullying in relation to appearance which might reasonably be expected to include weight or body size. Indeed, nine of the 124 policies mentioning appearance, also referred to weight-related keywords. However, appearance is a non-specific category which could include bullying in relation to physical identifiers of race, religion, health status, disability or any other physical characteristic (e.g. hair colour, spectacles etc.). Thirty-seven policies referred to appearance in relation to health, which may be a euphemistic way of referring to weight, but we are unable to infer this based on the data.

Research suggests specific wording matters in anti-bullying policies. In the US, Lessard and Puhl [43] found lower levels of weight bias (negative attitudes towards people with higher weight, as measured by the Fat Phobia scale [44]) among teachers when school district anti-bullying policies included mention of body weight; importantly, no association was found when policies mentioned appearance. Similarly, Lessard et al. [45] found lower levels of weight-related bullying in states where anti-bullying laws specifically refer to weight. By contrast, Hatzenbeuler et al. [46] found no relationship between state bullying laws mentioning “physical appearance” or “physical attributes” and weight-related bullying.

Ensuring weight is included in school anti-bullying policies could be a simple, yet important, step towards reducing rates of weight-related bullying. A recent review of anti-bullying policy interventions found clear evidence that inclusion of protected characteristics (such as sexual orientation or gender identity) was associated with lower levels of harassment, in part because of more frequent and more effective intervention by school staff [47].

However, while adding weight-related bullying into policies may be relatively simple, this alone will not be enough to tackle weight-related bullying [47, 48]; additional work will be needed to support implementation of the policy within the school environment. The YPAG members we spoke to felt that teachers in particular, as enforcers of school policy, would require specific training to overcome their own biases and feel confident in tackling weight-related bullying. A systematic review of 45 studies exploring weight bias in educational settings found evidence of implicit and explicit weight bias among training and in-service teachers, differential behaviour towards pupils according to their weight status, and the perception that pupils with obesity were less academically able [32]. Despite this, Puhl et al. [35] found most teachers recognise weight-related bullying as a problem and wish to intervene to address it. Importantly, however, two-thirds of teachers felt they needed additional training on how and when to do so [35]. Evidence suggests topic-specific training can be effective: for example, teachers who received LGBT-related training were more likely to intervene to support young people who identify as LGBT [49].

A further area highlighted by this study which warrants further investigation, is the differences between school type (mainstream state, private or special). Though it was rare even for private schools to include weight in their anti-bullying policies (17.5% did so), it is not clear why private schools would be three times more likely to do so than mainstream state schools, nor why none of the 67 special schools surveyed (whether state or privately funded) include mention of weight. Our data also tentatively (given the small cell counts) suggest that newer schools, such as academies and free schools, are more likely to include weight within their anti-bullying policies than those under Local Authority control. Further research to explore and understand these differences would be valuable.

### Strengths and limitations

This study surveyed anti-bullying policies from over 250 schools, including mainstream state, private and special schools. School data was collected from seven local authorities representing a range of urban and rural settings, and a population broadly representative of the UK population in terms of educational achievement and weight status. Young people were involved in both the study design and interpretation of findings.

This analysis was restricted to schools in one part of England; as can be seen in Table 1, however, the southwest region is close to the national average in terms of children’s academic achievement and prevalence of overweight. While we do not have local prevalence data for different forms of bullying, we have no reason to believe that the bullying policies of schools in the southwest would differ from those in other parts of England. Nonetheless, we cannot rule out the potential for regional differences in policy language and bullying dynamics. Future research should focus on more diverse national or internationals sample to strengthen findings and enable comparisons between regions and/or countries.

We created a comprehensive list of weight-related terms for policy searches, informed by the literature and initial pilot testing. While it is possible some terms may have been overlooked, we believe this is unlikely to affect the study’s conclusions. As highlighted in the Methods section, some policies were ambiguously worded and required us to judge the underlying intent– our approach was inclusive in these instances, thus lack of consideration of weight in school bullying policies may be greater than reported here. For some school-level characteristics, small cell sizes limited conclusions which could be drawn. Missing data could have influenced results, both for schools for which no policy was available, and through missingness on school characteristics which meant not all comparisons included all 255 schools.

Lastly, we note that our focus in this paper was specifically on weight-related bullying and school policies. It was beyond the scope of this work to examine the local prevalence or severity of systemic or other forms of discrimination in relation to school policies and their application. Nor have we considered intersectionality in relation to bullying and school policy. This is a complex field and the quantitative analysis we present here is an important first step in developing a comprehensive understanding.

### Recommendations

Based on these findings we propose three key recommendations. First, all schools should specifically include weight-related bullying in their anti-bullying policies. This would not only align schools’ policies with pupils’ lived experience by recognising the primary reported reason for school bullying, as shown by the evidence, but also provide a clear statement that harassment and discrimination based on a person’s weight is unacceptable and incompatible with the values of the school. Importantly, the term weight-related bullying would cover pupils with both under- and over-weight.

Second, materials to support policy implementation, including training for staff and pupils on recognising and challenging weight bias and discrimination, should be co-produced with input from the school community (pupils, teachers, governors, parents), researchers, and other relevant stakeholders (such as anti-bullying or mental health charities, health service providers).

Finally, the impact of specifically including weight in anti-bullying policies on levels of weight-related bullying in schools should be rigorously evaluated.

## Conclusion

Despite the frequency of weight-related bullying in schools, we found fewer than 7% of schools specifically mention weight in their anti-bullying policies. Schools should be encouraged to amend policies to specifically mention weight (rather than just appearance). Materials to help schools identify and respond to weight-related bullying should be developed in collaboration with the wider school community and relevant stakeholders, and their impact rigorously evaluated.

## Electronic supplementary material

Below is the link to the electronic supplementary material.

Supplementary Material 1

## Data Availability

The dataset supporting the conclusions of this article is available here https://data.bris.ac.uk/data/ [project DOI: 10.17605/OSF.IO/PFSB3].

## Abbreviations

BAME: Black, Asian and Minority Ethnic
BME: Black and Minority Ethnic
GCSE: General Certificate of Secondary Education
IMD: Index of Multiple Deprivation
ISI: Independent School Inspectorate
LA: Local Authority
LGBT: Lesbian, gay, bisexual and transgender
SEND: Special Educational Needs and Disabilities
UK: United Kingdom
US: United States
