# Supplementary material for: Weight-related bullying in schools: a review of school anti-bullying policies
Source: BMC Public Health. 2025 May 30;25:2006. doi: 10.1186/s12889-025-23170-9 (PMC12124066; doi:10.1186/s12889-025-23170-9)
Supplement: Supplementary file 1 — Supplementary Material 1 [file 12889_2025_23170_MOESM1_ESM.docx]

Supplementary tables

| **Supplementary table 1: school characteristics ^a^, mainstream state schools (N = 148)** | | | |
| --- | --- | --- | --- |
|  | **Category** | **N** | **%** |
| Type - detailed | Mainstream state: Academies | 122 | 82.4 |
|  | Mainstream state: Free schools | 13 | 8.8 |
|  | Mainstream state: LA maintained | 13 | 8.8 |
| Selective | Yes | 124 | 83.8 |
|  | No | 9 | 6.1 |
|  | *Missing* | 15 | 10.1 |
| Mixed or single sex | Mixed | 138 | 93.2 |
|  | Boys only | 4 | 2.7 |
|  | Girls only | 6 | 4.1 |
| Age range of pupils | 11+ | 143 | 96.6 |
|  | Earlier | 5 | 3.4 |
| Ofsted Rating | Outstanding | 17 | 11.5 |
|  | Good | 75 | 77.6 |
|  | Requires improvement | 12 | 8.1 |
|  | Serious weaknesses | 1 | 0.7 |
|  | Special measures | 2 | 1.4 |
|  | *Missing* | 5 | 3.4 |
| Progress8 | Well above average | 20 | 13.5 |
|  | Above average | 29 | 19.6 |
|  | Average | 64 | 43.2 |
|  | Below average | 12 | 8.1 |
|  | Well below average | 13 | 8.8 |
|  | *Missing* | 10 | 6.8 |
| Type of policy | Bullying | 123 | 83.1 |
|  | Behaviour | 24 | 16.2 |
|  | *Missing* | 1 | 0.7 |
| Policy set at school or trust level | School | 119 | 80.4 |
|  | Trust | 29 | 19.6 |
|  | **Median (IQR)** | **Range** | *N missing* |
| % pupils eligible for free school meals | 17.7  (13.0-24.8) | 2.2–59.9 | 3 |
| IMD decile ^b^ | 7 (4–9) | 1–10 | 0 |
| ^a^ School background data obtained from https://www.compare-school-performance.service.gov.uk/ and https://www.isc.co.uk/. Policy data were obtained from schools’ individual websites.  ^b^ Based on schools’ Lower Super Output Area | | | |

| **Supplementary Table 2: school characteristics ^a^, private schools only (N = 40)** | | | |
| --- | --- | --- | --- |
|  | **Category** | **N** | **%** |
| Mixed or single sex | Mixed | 33 | 82.5 |
|  | Girls only | 7 | 17.5 |
| Age range of pupils | 11+ | 11 | 27.5 |
|  | Starts earlier | 29 | 72.5 |
| Independent Schools Inspectorate (ISI) rating – academic attainment | Excellent | 26 | 65.0 |
|  | Good | 7 | 17.5 |
|  | Missing | 7 | 17.5 |
| Independent Schools Inspectorate (ISI) rating – personal development | Excellent | 30 | 75.0 |
|  | Good | 3 | 7.5 |
|  | Missing | 7 | 17.5 |
| Type of policy | Bullying | 37.0 | 92.5 |
|  | Behaviour | 3.0 | 7.5 |
| **^a^** School background data obtained from https://www.compare-school-performance.service.gov.uk/ and https://www.isc.co.uk/. Policy data were obtained from schools’ individual websites. | | | |

| **Supplementary Table 3: school characteristics ^a^, state special schools only (N = 41)** | | | |
| --- | --- | --- | --- |
|  | **Category** | **N** | **%** |
| Mixed or single sex | Mixed | 40 | 97.6 |
|  | Boys only | 1 | 2.4 |
| Age range of pupils | 11+ | 5.0 | 12.2 |
|  | Earlier | 36.0 | 87.8 |
| Ofsted Rating | Outstanding | 7 | 17.1 |
|  | Good | 26 | 63.4 |
|  | Requires improvement | 4 | 9.8 |
|  | *Missing* | 4 | 9.8 |
| Progress8 | Well below average | 12 | 29.3 |
|  | *Missing* | 29 | 70.7 |
| Type of policy | Bullying | 38 | 92.7 |
|  | Behaviour | 3 | 7.3 |
|  | **Median (IQR)** | **Range** | *N missing* |
| % pupils eligible for free school meals | 48.3(40.8–62.9) | 27.4–76.2 | 7 |
| **^a^** School background data obtained from https://www.compare-school-performance.service.gov.uk/ and https://www.isc.co.uk/. Policy data were obtained from schools’ individual websites. | | | |

| **Supplementary Table 4: school characteristics ^a^, private special schools only (N = 26)** | | | |
| --- | --- | --- | --- |
|  | **Category** | **N** | **%** |
| Mixed or single sex | Mixed | 26 | 100 |
| Age range of pupils | 11+ | 6 | 23.1 |
|  | Earlier | 20 | 76.9 |
| Type of policy | Bullying | 24 | 92.3 |
|  | Behaviour | 2 | 7.7 |
| **^a^** School background data obtained from https://www.compare-school-performance.service.gov.uk/ and https://www.isc.co.uk/. Policy data were obtained from schools’ individual websites. | | | |
